# Surgical management of paediatric pelvic fractures: a prospective case series and early experience from a level one Egyptian trauma centre

**DOI:** 10.1007/s00264-022-05509-8

**Published:** 2022-07-23

**Authors:** Mohamed Arafa, Ahmed A. Khalifa, Ali Fergany, Mostafa A. Abdelhafez, Aly Mohamedean, Faisal Fahmy Adam, Osama Farouk

**Affiliations:** 1 Orthopaedic Department, Assiut University Hospital, Assiut, Egypt; 2 Orthopaedic and Traumatology Department, Qena Faculty of Medicine and University Hospital, South Valley University, Kilo 6 Qena-Safaga Highway, Qena, Egypt

**Keywords:** Paediatric, Fracture, Pelvic ring injury, Fracture pelvis

## Abstract

**Purpose:**

We aimed to report our early experience treating paediatric pelvic fractures (PPF) surgically, reporting on indications, outcomes, and complications.

**Methods:**

Patients aged 0–15 with PPF treated surgically at a level I trauma centre were included prospectively between 2016 and 2018. Fractures were classified according to AO/OTA classification system. Functional evaluation was performed using a modification of the Majeed functional scoring system. Radiological evaluation of vertical and posterior displacement was performed according to Matta and Tornetta criteria and the method described by Keshishyan et al. for assessing pelvic rotational asymmetry.

**Results:**

We included 45 patients (77.8% males and 22.2% females), with a mean age of 9.53 ± 3.63 and 19.87 ± 8.84 months of mean follow-up. The functional outcome was excellent in 42 (93.3%) patients, good in two (4.4%), and fair in one (2.2%). Radiologically, the vertical displacement improved from 5.91 ± 4.64 to 3.72 ± 2.87 mm (*p*-value 0.065), the posterior displacement improved from 7.87 ± 8.18 to 5.33 ± 13.4 mm (*p*-value 0.031), and the symphyseal diastasis improved from 9.88 ± 7.51 mm to 7.68 ± 3.18 mm (*p*-value 0.071). Residual pelvic asymmetry improved from 1.2 ± 0.61 to 0.8 ± 0.7 (*p*-value 0.001). Complications occurred in 21 (46.7%) patients, 11 (24.4%) pin tract infection, six (13.3%) limb length discrepancy, two (4.4%) prominent metals, one (2.2%) subcutaneous haematoma, one (2.2%) infected ISS.

**Conclusions:**

We achieved acceptable functional and radiological outcomes after surgically treating a group of patients with PPF, which was relatively safe with minimal complications. The proper approach and fracture fixation tool should be tailored according to the fracture classification and the presence of associated injuries.

**Supplementary Information:**

The online version contains supplementary material available at 10.1007/s00264-022-05509-8.

## Introduction 

According to various reports, pediatric pelvic fractures (PPFs) represent from 1.6 to 20% of patients presented with pelvic fractures [1–4]; it entails a significant concern owing to the vulnerability of the paediatric population and the possible long-term sequel [5, 6]. It differs from adult pelvic injuries since the immature paediatric skeleton has inherent flexibility due to lax sacroiliac joints (SIJs) and symphysis pubis; furthermore, the cartilaginous cover acts as a shock absorber [7, 8]. Hence, a child presenting with a pelvic fracture indicates a significant trauma [1].

Nonsurgical management has been the standard of care in PPF, considering that the paediatric skeleton can remodel; however, this led to various long-term complications, including malunion, nonunion, scoliosis, and pelvic asymmetry [9–12]. Up to 30% of paediatric patients with unstable pelvic fractures suffered from limping, residual pain, low back pain, and permanent neurological damage [5, 13].

The decision to nonsurgically or surgically treat these fractures depends on several variables: first, the patient’s physiological age and general status, as well as any associated skeletal or nonskeletal injuries; second, the nature of the fracture (classification and displacement); third, if the surgical option was chosen, would it be on an emergency basis as part of the damage control or would it be preferably postponed to be performed within the first few days after trauma [5, 9, 14].

Although the principles of surgical management (resuscitation, provisional fixation, and definitive fixation) apply among various reports on PPF, no definite guidelines or consensus for surgical PPF management have been developed [14], which could be attributed to the rarity of these injuries and studies with small sample sizes [1, 15]. Data related to PPF management are lacking in our areas (the Middle East and North Africa). Hence, this study aimed to report our early experience in PPF surgical management, focusing on the indications, functional and radiological outcomes, and complication incidence.

## Materials and methods

A prospective case series study at the pelvis and acetabulum trauma unit in a regional Egyptian academic level one trauma centre was performed between the 1st of January 2016 and the end of December 2018. A total of 62 patients with PPF (as a sole injury or associated with other injuries) were admitted to the trauma unit during this period; we included surgically treated skeletally immature patients (up to 15 years old) who presented with open or closed pelvic fractures with or without associated injuries who were admitted to the hospital within seven days of trauma. Patients with pathological fractures, a late presentation of > one week, endocrinal disturbance, unfit for surgery, parents refusing surgery or conservatively treated, and those with an incomplete record at last follow-up were excluded from the study. After excluding 17 conservatively treated patients, 45 surgically treated patients were eligible for inclusion.

This series followed a uniform treatment protocol for all patients who presented with a suspected PPF. Initial assessment at the presentation time to the trauma unit was performed according to the ATLS protocol. Upon completing the primary survey, urgent pelvic fracture stabilization was performed using a pelvic binder. Radiographic assessment was initially performed using pelvic anteroposterior (AP), inlet, and outlet radiographic views. If a displaced fracture is suspected, pelvic computed tomography (CT) with 3-mm cuts was performed as a part of the pre-operative assessment and planning (the parents or caregivers were informed regarding the significance and possible hazards of radiation exposure, and informed consent was obtained for all patients) [5]. All patients were assessed using a specified fractured pelvis and acetabulum registry form sheet developed in our unit (Supplementary file 1).

**Fig. 1:**
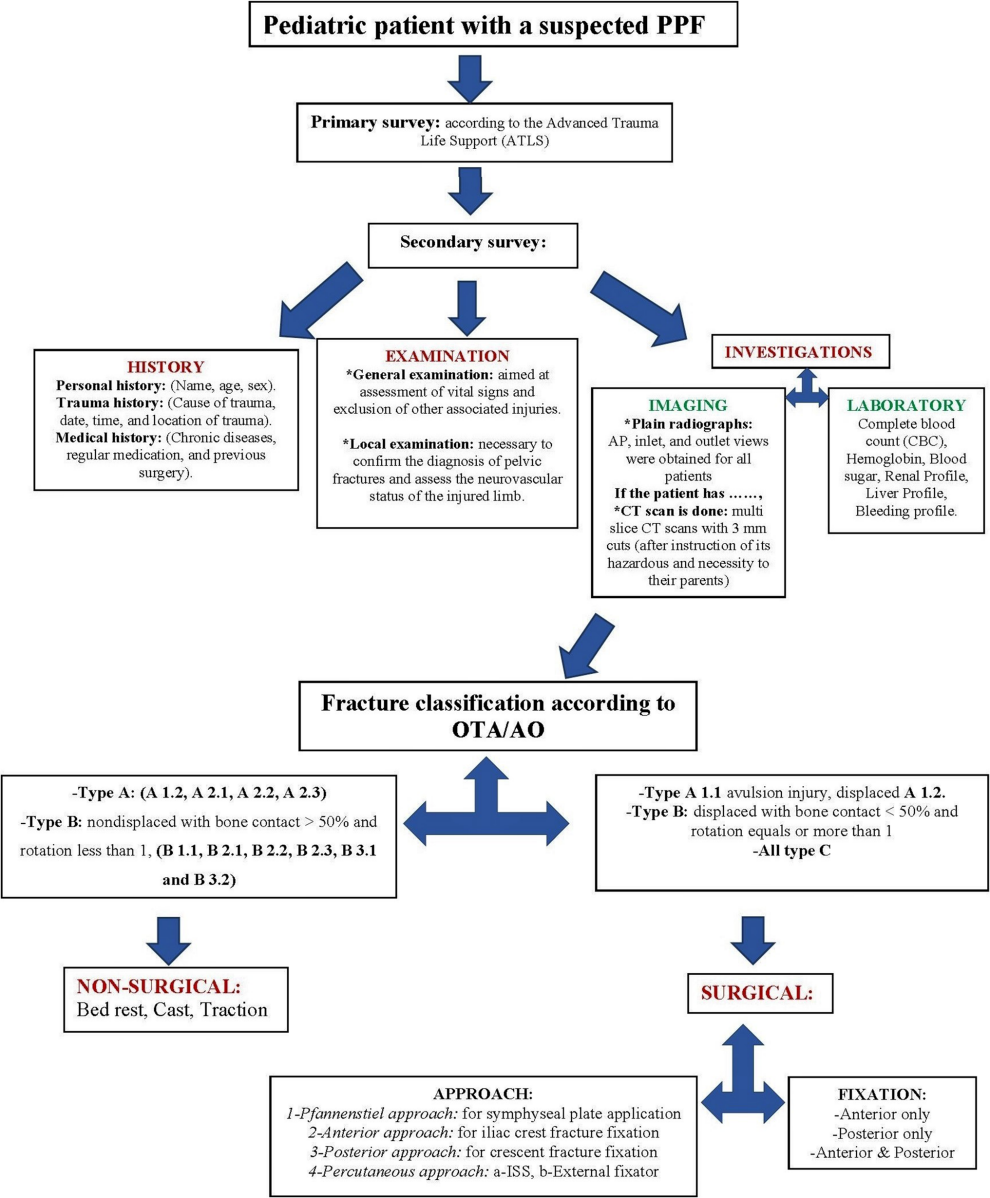
A flow chart diagram showing PPF patient’s pathway according to our department policy

Fracture classification was based on the modified Tile AO/OTA classification, dividing pelvic fractures into three basic types according to posterior sacroiliac complex stability and integrity. Type A pelvic fractures do not involve the posterior arch (stable), whereas type B results from rotational forces that cause partial posterior complex disruption (rotationally unstable). In type C, there is complete posterior complex disruption (rotationally and vertically unstable) (Table 1) [16–19]. Fractures classified as A 1.1 avulsion injury, displaced A 1.2 and A 2.2 (if displacement is more than 1.5 cm), type B with a significant rotational displacement of the pelvic ring and loss of bone contact at the fracture site, and all type C pelvic fractures were surgically treated. Following standards of care in our institute, the patients’ pathway is shown in Fig. 1.

**Table 1:** Fractures classified according to modified Tile OTA/AO classification system

Type		Sub-types	Description	Current study fractures classifications-Total 45 (100%) patients^a^
**A** (Rotationally and vertically stable, posterior arch is intact)	1 (avulsion fractures)	**A 1.1**	Iliac spine avulsion	1 (2.2%)
		**A 1.2**	Iliac crest apophyseal avulsion	4 (8.9%)
		**A 1.3**	Ischial tuberosity avulsion	0 (0.0%)
	2 (fractures of the innominate bone)	**A 2.1**	Fracture iliac bone	0 (0.0%)
		**A 2.2**^**b**^	Unilateral fracture pubic rami	1 (2.2%)
		**A 2.3**	Bilateral fracture pubic rami	0 (0.0%)
	3 (transverse fractures of the sacrum caudal to S2)	**A 3.1**	sacrococcygeal dislocation	0 (0.0%)
		**A 3.2**	un-displaced fracture sacrum	0 (0.0%)
		**A 3.3**	displaced fracture sacrum	0 (0.0%)
**B** (Rotationally unstable, vertically stable, incomplete disruption of the posterior arch)	1 (incomplete disruptions (external rotation)	**B 1.1**^**c**^	Open book through anterior sacroiliac joint	5 (11.1%)
		**B 1.2**	Open book with sacral fracture	0 (0.0%)
	2 (incomplete disruptions (internal rotation)	**B 2.1**	Lateral compression with anterior sacral compression injury	2 (4.4%)
		**B 2.2**	Partial fracture subluxation of sacroiliac joint	11 (24.4%)
		**B 2.3**	incomplete posterior iliac wing fractures	0 (0.0%)
	3 (incomplete disruption (bilateral)	**B 3.1**	Open book through bilateral anterior sacroiliac joint	1 (2.2%)
		**B 3.2**	Internal rotation with contralateral external rotation injury	2 (4.4%)
		**B 3.3**	bilateral internal rotation injuries	0 (0.0%)
**C** (Rotationally and vertically unstable, complete disruption of the posterior arch)	complete disruptions which can be unilateral or bilateral	**C 1.1**	Vertical shear through the iliac wing	8 (17.8%)
		**C 1.2**	Vertical shear through sacroiliac joint	7 (15.6%)
		**C 1.3**	Vertical shear through the sacrum	3 (6.7%)

^a^Data presented as numbers and percentages

^b^Gapped superior and inferior pubic rami

^c^Symphyseal gap was more than 2.5 cm

### Operative management

The mean time from admission to surgery was 3.62 ± 2.41 (range, 1–11) days. Informed written consent, including details of the surgical procedure, benefits, possible risks, and complications, was obtained from the parents of all participating patients.

All patients were operated on under general anaesthesia, and surgical approaches were determined according to the fracture classification, including percutaneous, Pfannenstiel approach, direct anterior approach (for iliac crest fracture fixation), and direct posterior approach (for crescent fracture fixation).

Patients were positioned supine (Pfannenstiel approach and direct anterior approach) and prone (direct posterior approach) on a radiolucent table, allowing clear fluoroscopic visualization of the AP, inlet, and outlet radiographic projections. The injured limb was draped free with the knee in a flexed position to relax the iliopsoas and external iliac/femoral NV bundle. A Foley catheter was inserted for bladder drainage, protection, and fluid balance monitoring. All patients had received one dose of first-generation cephalosporin after dose adjustment based on body weight and age within one hour pre-operatively.

### Surgical techniques

**Fig. 2:**
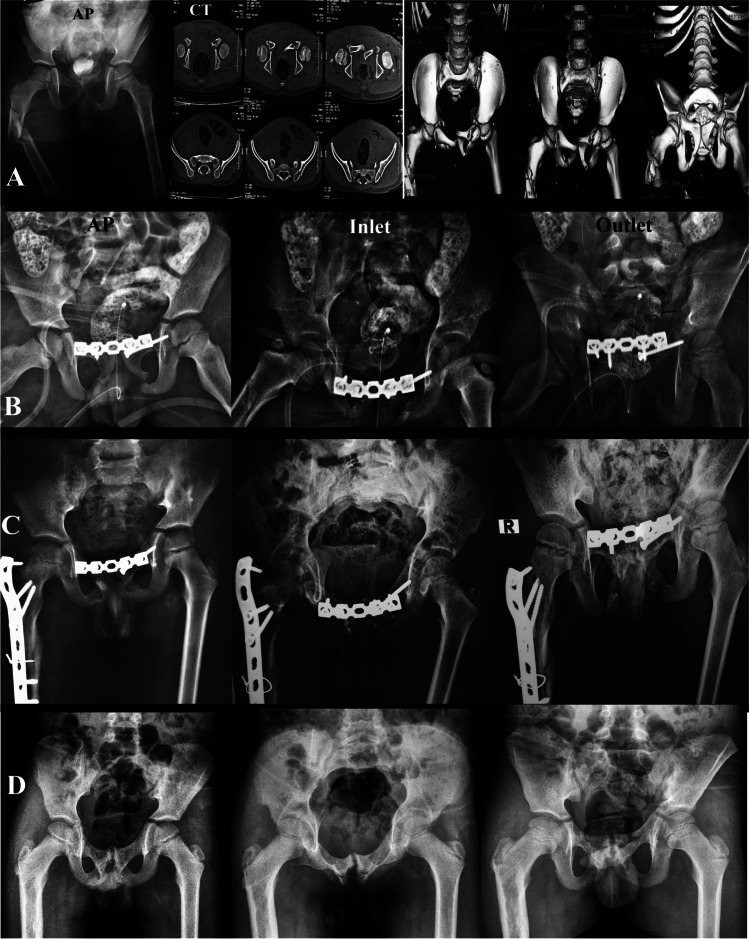
Male child 8 years old presented after a motor car accident with fracture pelvis type B2.2 (Lt crescent fracture, anterior symphyseal disruption, Lt pubic rami fracture) treated with a symphyseal plate. Associated injuries (fracture Rt femur, urethral injury) ORIF of the femoral fracture was performed in another session. **A** Preoperative imaging studies (plain AP pelvis radiograph and a CT scan). **B** Immediate post pelvic fracture fixation. **C** After 15 months of follow-up. **D** After 29 months of follow-up (hardware was removed)

**Fig. 3:**
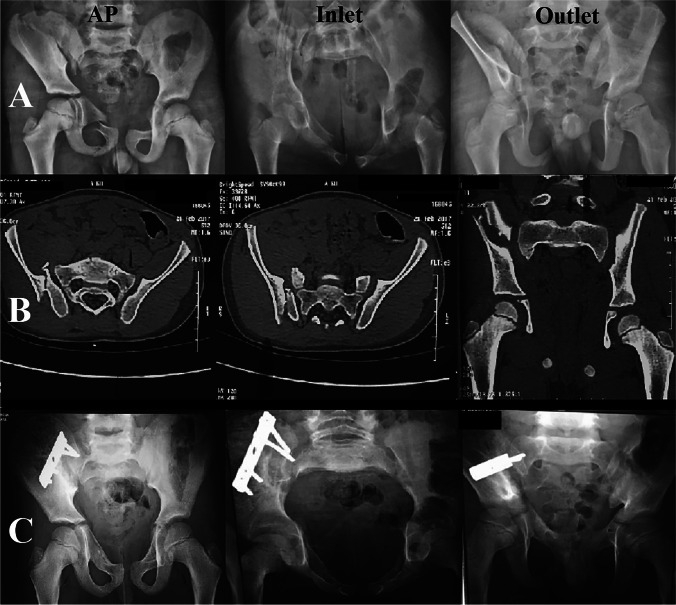
Male child 8 years old presented after fell from height with fracture pelvis type C 1.1 (vertical shear through Rt iliac bone, and Rt superior and inferior pubic rami) treated by posterior plating. Preoperative imaging: **A** plain radiographs and **B** CT scan. **C** plain radiographs after 14 months of follow-up

**Fig. 4:**
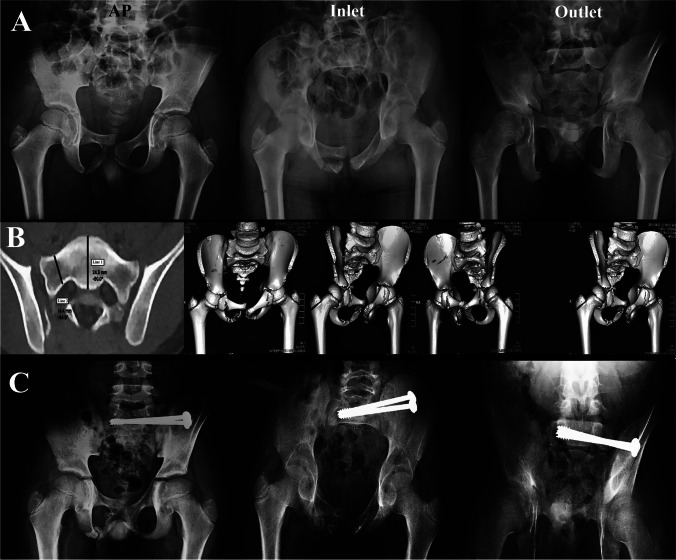
Male child 10 years old presented after a motor car accident with fracture pelvis type C1.2 (with Lt sacroiliac dislocation) treated by iliosacral screws. **A** Preoperative plain radiographs. **B** Preoperative CT scan showing how to plan the size of screws to be used for fixation. **C** Plain radiographs after 24 months of follow-up

1. *Pfannenstiel approach* was used in two (4.4%) patients to place a symphyseal plate for anterior pelvic disruption. Fixation was supplemented by an iliosacral screw (ISS) in one patient. We used a reconstruction plate of 3.5 in one patient and 4.5 in the other, placed “on top” of the symphysis (Fig. 2).
2. *Direct anterior approach for iliac crest* was used in four (8.9%) patients, of whom two had bilateral displaced iliac apophysis, and two had unilateral displaced iliac apophysis. In all patients, fixation and reattachment of the displaced segment were performed using transosseous sutures (Vicryl 2); Kirschner-wire was used for fixation augmentation in one patient. A mini-open direct approach over the anterior inferior iliac spine (AIIS) was used to fix an avulsion fracture using a lag screw.
3. *Posterior approach for crescent fracture* was used in seven (15.6%) patients, of whom six underwent posterior plating, and one had a small posterior fragment of the crescent element fixed by a posterior lag screw to close the fracture gap and ISS to close the SIJ. We used a 3.5-, 4.5-, or 6.5-mm lag screw according to the bone size; subsequently, a contoured plate was used as a neutralization plate (small DCP 3.5 mm, four or five holes), and one or two plates were used according to the fracture pattern (Fig. 3).
4. *Percutaneous approach*
  - ISS fixation alone was used in 19 (42.2%) patients and transiliac-transsacral screw in a ten year-old patient. ISS is used for fixing SIJ dislocation or sacral fractures, some types of crescent fractures, and injuries to the Risser’s growth nuclei. Partially threaded cannulated lag screws (7.3 or 4.5 mm) with a washer were used for SIJ compression. We used axial CT images to predict the screw size, matching the size of the S1 body by measuring its AP diameter and the AP diameter of the sacrum ala (Fig. 4).
  - Anterior external fixator alone was used in six (13.3%) patients as a definitive fixation method. We used Schanz screws with 4- or 5-mm diameter in the supraacetabular region by using the AIIS as a starting point.
  - Combined ISS and anterior external fixator were used in five (11.1%) patients. A threaded wire was used in one patient (a 4-year-old male presented with a type C fracture) instead of the ISS.

### Post-operative and follow-up protocol

During the hospital stay, an immediate post-operative neurovascular evaluation was performed. Radiographic assessment of fracture reduction quality and implant positioning was performed using plain pelvic AP, inlet, and outlet radiographic views.

Follow-up visits were scheduled at two weeks for wound assessment and suture removal, six weeks for radiological and functional assessment, then at three, six and 12 months, and annually thereafter. All patients were allowed to start weight-bearing after six weeks if pelvic fracture union radiological evidence was noted.

Assessment during follow-up visits was performed as follows:

1. *Functional evaluation*: using a modification of the Majeed functional scoring system [20] that includes 30, 10, 36, 12, and 12 points for pain, sitting, standing, gait unaided, and walking distance, respectively, we excluded four points for sexual intercourse and modified the 20 points for work as shown in Table 2. The total score was 96; scores of > 70, 55–69, 45–54, and < 45 were graded as excellent, good, fair, and poor, respectively.
2. *Radiological evaluation:* AP, inlet, and outlet plain pelvic radiographs were performed.
  A- For assessing vertical (AP view) and posterior displacement (inlet view), the Matta and Tornetta criteria [21, 22] were used (excellent, good, fair, and poor when a residual displacement of ≤ 4 mm, 5–10 mm, 11–20 mm, and > 20 mm, respectively).
  B- For assessing pelvic rotational asymmetry, we used the method described by Keshishyan et al. [10, 23] in the AP view, including calculating the deformity index. Normal anatomical variation in this measurement is considered up to 4 mm of asymmetry, which may occur due to pelvic rotation when obtaining the radiographs. If > 5 mm of asymmetry, the deformity is considered pathological. The amount of symphyseal diastasis was assessed in the AP view as well.
  C- Fracture union assessment: evidence of radiological union was defined as anterior and posterior cortical bridging, and the absence of fracture lines of the pelvic ring in the follow-up radiograph was correlated with improvement in clinical pain.
3. *Complications* were documented and reported at various points (peri-operative, post-operative, and at any follow-up visit).

**Table 2:** Modifications on work category of Majeed score

Total 20 points	Original category by Majeed	Modification
0—4	No regular job	Did not return back to activity/school
8	Light work	Marked limitation of sports activity/playing
12	Change of job	Repeated absence from school/sport training
16	Same job, reduced performance	Return back to activity, reduced performance
20	Same job, same performance	Return back to full activity, pre-fracture status

### Statistical analyses

Statistical analysis was performed using the SPSS version (International Business Machines, Chicago, IL) (version 16 windows). Normally distributed continuous data were reported as mean ± standard deviation. Such data were compared with Student’s *t*-test data in the case of two groups and ANOVA test followed by post hoc analysis in more than two groups. Results were considered statistically significant at a *p*-value less than or equal to 0.05.

## Results

Of the 45 patients included in this study, 35 (77.8%) were males, with a mean age of 9.53 ± 3.63 (range, 5–15) years. Nineteen (42.2%), 13 (28.9%), and four (8.9%) had open, narrowed, and closed triradiate cartilages, respectively. The mean duration of hospital stay was 7.67 ± 5.07 (range, 3–27) days. The mean operative time was 36 ± 14.2 (range, 20–80) min, and 7 (15.6%) patients required blood transfusion. Further details of the age groups, trauma mechanism, associated injuries, and fixation methods are shown in Table 3. All patients were available for assessment at the mean last follow-up visit of 19.87 ± 8.84 (range, 12–36) months.

**Table 3:** Characteristics of the study group, 45 (100%) patients

Age groups (years)^a^			
< 6		9 (20%)	
6–10		16 (35.6%)	
11–15		20 (44.4%)	
Cause of trauma^a^			
Motor car accidents		17 (37.8%)	
Pedestrian accidents		11 (24.4%)	
Fell downstairs		7 (15.6%)	
Fell on the ground		6 (13.3%)	
Fell from height		4 (8.9%)	
Associated injuries^a^			
Lower limb injuries		11 (24.4%)	
Internal hemorrhage (one patient had a splenic tear^b^)		6 (13.3%)	
Upper limb injury		6 (13.3%)	
Bladder injury, urethral injury, perineal injuries		4 (8.9%)	
Acetabulum fractures		2 (4.4%)	
Spine injury		1 (2.2%)	
Lumbo-sacral plexus injury		1 (2.2%)	
Head injury		1 (2.2%)	
Morel-Lavallee lesion		1 (2.2%)	
Total		33 (73.3%)	
Methods of fixation			
Fixation tools	Fracture classification^a^		Operative time *(in minutes)*^c^
*A- Anterior fixation only, 12 patients:*			
External fixator	-A 2.2 -B 1.1 (3), B 3.1 -C 1.2	6 (13.3%)^d^	29.2 ± 7.4 (20:40)
Lag screws for AIIS	-A 1.1	1 (2.2%)	25
Symphyseal plate	-B 2.2	1 (2.2%)^d^	60
Trans osseous sutures	-A 1.2	4 (8.9%)	25 ± 4.1 (20:30)
*B- Posterior fixation only, 26 patients:*			
Ilio- iliac screw	-C 1.2	1 (2.2%)	40
ISS	-B 1.1, B 2.1, B 2.2 (9), B 3.2 -C 1.1, C 1.2 (3), C 1.3 (3)	19 (42.2%)	30.3 ± 7 (20:45)
Posterior plating	-C 1.1	6 (13.3%)	37.5 ± 5.2 (30:45)
*C- Combined anterior and posterior fixation, 7 patients:*			
ISS, external fixator	-B 1.1, B 2.2, B 3.2, -C 1.1, C 1.2	5 (11.1%)	59.2 ± 8.6 (45:70)
ISS, symphyseal plate	-B 2.1	1 (2.2%)	80
Threaded wires, external fixator	-C 1.2	1 (2.2%)	55

^a^Data presented as numbers and percentages. ISS iliosacral screws, AIIS anterior inferior iliac spine

^b^Patient underwent emergency surgical abdominal exploration and splenectomy

^c^Data presented as mean ± SD

^d^One external fixator and one symphyseal plate were applied as an emergency procedure on the same day of admission

**The functional outcome** was excellent, good, and fair in 42 (93.3%), 2 (4.4%), and 1 (2.2%) patients, respectively, according to the modification of the Majeed functional scoring system. The functional score decreases with associated injuries and increased length of hospital stay (Table 4).

**Table 4:** Difference in functional outcomes according to various demographic factors

Parameters		Functional outcome	
		Mean ± SD	*p*-value^a^
Age: (years)	< 6	95.2 ± 2.3	0.331
	6–10	93.1 ± 5.3	
	11–15	89.7 ± 13.8	
Sex:	Male	91 ± 11	0.234
	Female	95.3 ± 2.2	
Hospital stay: (days)^b^	< 5 days (10 patients, 22.2%)	95.3 ± 2.2	p1 = 0.47 **p2 = 0.04** **p3 = 0.02**
	5 ≤ 10 days (26 patients, 57.8%)	93.4 ± 8.1	
	> 10 days (9 patients, 20%)	84.2 ± 15.7	
Time between admission and surgery: (days)	< 3 (18 patients, 40%^**c**^)	91.4 ± 10.8	0.819
	3–6 (22 patients, 48.9%)	91.9 ± 10.4	
	7–11 (5 patients, 11.1%)	94.6 ± 3.1	
Fracture pattern	Type A	92.8 ± 9.3	0.589
	Type B	92.3 ± 10.6	
	Type C	88.7 ± 12.4	
complications	Yes	90.3 ± 8.3	0.409
	No	92.9 ± 10.7	
Associated injuries	Yes	88.7 ± 13.4	**0.030**
	No	95.1 ± 2.4	

^**a**^Student’s *t*-test was used to compare quantitative variables between two groups and ANOVA test followed by post hoc analysis for more than two groups

^**b**^[p1 (< 5 days vs. 5 ≤ 10 days), p2 (< 5 days vs. > 10 days), p3 (5 ≤ 10 days vs. > 10 days)]

^**c**^Two patients were operated upon on the same day of admission

**For the radiological outcomes**, improvement in different parameters is shown in Table 5; for the 18 patients with type C fracture, Matta and Tornetta’s grading system evaluation was as follows: three patients showed an excellent reduction in the vertical displacement, and 15 had a good reduction. In contrast, seven patients showed an excellent reduction in the posterior displacement, good in ten, and poor in one.

**Table 5:** Radiological outcome

Parameter	Preoperative^a^	Postoperative^a^	*p*-value^b^
1-Vertical migration (mm)	5.9 ± 4.6 (0.0 to 17.6)	3.7 ± 2.9 (0.0 to 8.7)	0.065
2-Posterior displacement (mm)	7.9 ± 8.2 (0.0 to 35.0)	5.3 ± 13.4 (0.0 to 57.8)	**0.031**
3-Symphyseal diastasis (mm)	9.9 ± 7.5 (-3.4 to 44.5)	7.7 ± 3.2 (2.7 to 18.2)	0.071
4-Asymmetry (rotation)	1.2 ± 0.6 (0.1 to 2.4)	0.8 ± 0.7 (0.0 to 3.8)	**0.001**
5-Deformity index	0.7 ± 0.4 (0.0 to 2)	0.5 ± 0.3 (0.0 to 1.0)	**0.001**

^a^Data presented as Mean ± SD (range)

^b^Student’s *t*-test

1 and 2 were measured in 18 patients classified as type C (according to Matta and Tornetta grading system [21, 22]). 3 was measured in all patients, and 4 and 5 were measured in 39 patients classified as type B and C (according to the method of Keshishyan et al. [10, 23])

**Complications** were reported in 21 (46.7%) patients. No intra-operative complications or mortality was related to the injury or surgical management. One (2.2%) patient developed a subcutaneous hematoma at the contralateral side of the injury after ISS fixation, conservatively managed. Pin tract infection developed in 11 (24.4%) patients; all of them improved after external fixator removal and daily dressing without the need for surgical debridement. One (2.2%) patient had an infected ISS after 8 months post-operatively (for crescent fracture fixation), which necessitated metal removal. Superior gluteal injury occurred once during ISS removal. In the last follow-up visit, six (13.3%) patients had residual vertical migration with a limb length discrepancy (LLD) of < 1 cm (range, 4.2–8.7 mm). Prominent metals were reported in two (4.4%) patients (one had posterior plating for a crescent fracture, and the other had symphyseal plate); metal removal was performed for both.

## Discussion

Anatomically, the immature pediatric patient skeleton offers better flexibility with greater joint (sacroiliac and symphysis pubis) laxity and abundant cartilage, which allows for superior shock absorption and an increased remodeling potential [7, 24]. These inherent characteristics of the paediatric patients’ pelvis encouraged more nonsurgical lines for PPF management, including bed rest, traction, pelvic slings, or hip spica casts [12, 25–27]. PPF treatment recommendations have changed during the last decade, with more surgeons suggesting surgical intervention [1, 15, 28], especially in unstable fracture patterns, to avoid late comorbidities associated with nonsurgical management options, including low back pain, LLD, early sacroiliac fusion, iliac wing, and hemipelvis undergrowth, which have been reported in up to 30% of the patients with PPF [13, 26, 27, 29, 30].

The cutoff age limit to classify a patient with pelvic trauma as “paediatric” is variable among studies; Gansslen et al. identified an upper age limit between 14 and 20 years old [3], whereas Eisa et al. considered the cutoff age for the pediatric group as 16 years old [4]. Others considered the triradiate cartilage status, Hermans et al. [2] defined a range for triradiate cartilage fusion from 13 to 16 years old; hence, some authors considered this a watershed age for changing pelvic injury patterns and management strategies from those designed for children to those adapted and more relevant in adults. We included patients up to 15 years old to avoid this age debate, with only four patients having a closed triradiate cartilage.

Although some surgeons suggested that older children (especially with closed triradiate cartilage) could be managed according to criteria applied to adults [31], deciding to treat younger children with PPF surgically depends on various factors, including fracture classification, the general status of the patients, surgical team efficiency, and whether the surgery should be performed as an emergency measure (damage control) or to restore pelvic ring anatomy to prevent later complications [19, 27, 30].

We used the AO/OTA classification system in the current series, which mainly considered fracture stability and displacement; we built our surgical decision partially on this classification system, as other factors should be taken into consideration. Regarding fracture pelvis type A which is known to be inherently stable and amenable to conservative management, however, in some situations, surgical management was mandatory; we surgically treated six patients with type A fractures (5 had avulsion fractures, and one had pubic rami fractures), if the avulsed fragment which is usually under the pull of muscle forces is displaced or bigger than 1.5 cm, surgical fixation is preferable through a direct approach using lag screws, or reattachment using sutures as in the case of A 1.2 fractures (Iliac crest apophyseal avulsion).

The benefit of surgical fixation of these stable injuries was shown in a systematic review by Calderazzi et al., who investigated the outcomes of treating pelvic avulsion fractures conservatively or surgically; the authors found that patients treated surgically had better functional outcomes, faster return to sports activities, and less incidence of fracture nonunion [32]. For the iliac crest apophyseal avulsion injuries, Li et al. presented their experience of managing ten patients surgically; the authors reported that their patients were able to engage immediately in active rehabilitation 2 days postoperatively, and they recovered full athletic activities in 4 weeks after the injury, and no complications were reported in their series [33]. Furthermore, Pogliacomi et al. reported that half of their conservatively treated adolescent patients who presented with iliac avulsion fractures suffered from painful hip impingement caused by excessive new bone formation from the healing process [34].

For types B and C, treating these injuries surgically is a more straightforward decision to make than for type A, as those fractures are usually displaced and unstable; however, it is more challenging to decide on anterior, posterior, or both sides fixation which depends mainly on the fracture configuration and intraoperative stability after preliminary reduction and fixation, in our experience, most of the patients (84.4%) needed only either anterior or posterior fixation.

Another factor we considered in deciding on surgical management is the presence of concomitant injuries; in our series, one patient presented with type A 2.2 (gapped unilateral fracture of the superior and inferior pubic rami) had a concomitant femoral and tibial shaft fractures, which were treated by ORIF and a cast respectively; for a better nursing and patient rehabilitation, we decided to stabilize the pelvis using an external fixator. Another example of the importance of concomitant injury consideration when making a treatment decision is patients presented with type B 2.1 and B 2.2 fractures with an associated urethral and bladder injuries; in order to create a stable foundation for the bladder to be repaired and to heal, we had to fix the fracture before the urosurgery team performs their repair.

For selecting the surgical approach, we believe that the injury location and either posterior, anterior, or both sides fixation are decided that dictate approach selection; hence, we used different approaches (direct anterior, anterior Pfannenstiel, direct posterior, and percutaneous approaches).

Tile et al. offered implant options according to patient age and based on the AO/OTA classification system [27]. In this study, we used various fixation tools through open or percutaneous approaches based mainly on the fracture classification and injury site according to Tile et al.; however, we had to modify and decide the appropriate fixation tool based on bone geometry in some patients, and the decision could be changed intra-operatively based on the surgeon decision. We used large and small set plates and screws for symphyseal disruption and crescent fracture fixation; for avulsion fractures, we used a combination of transosseous sutures and k-wires; however, for an AIIS avulsion with a larger fragment, we used a lag screw. To select the most suitable screw size for ISS, we calculated the propel size based on S1 diameter in the axial CT images. Furthermore, the sizes of the Schanz screws used for external fixation vary from 4 to 5 mm, which is dependent mainly on the bone geometry and the feel of construct stability intra-operatively.

The inconsistency of fixation tools had also been reported in previous studies; a systematic review by Sridharan et al. [9] included 10,132 PPF, 8.8% were surgically treated. The most common surgical intervention was open reduction and internal fixation (ORIF) with or without ISS fixation (83.6%), followed by percutaneous screw fixation alone (7.2%) and external fixation with or without ISS (5.2%). The study by Zhu et al. included 40 patients with a mean age of 5.9 ± 3.1 (range, 2–14.5) years; although all the patients were diagnosed with unstable PPF, they were treated with an external fixator alone; however, patients diagnosed with fracture type C (22 patients) had post-operative traction to control the vertical element [11]. So, we believe that the area of choosing the best fixation tool still needs further investigation.

For functional assessment, we used a modification of the Majeed functional scoring system [20] since it is specific for pelvic fractures, and we achieved excellent outcomes in 93.3% of our patients; however, it was not frequently utilized in PPF outcome assessment which made it difficult to compare our results with previous reports. Signorino et al. [35] and Zhu et al. [11] used the more general WeeFIM instrument (formerly known as the Functional Independence Measure for Children), which assesses self-care, mobility, and cognition. In a recent study by Fahmy and Abdelmoneim, including 21 surgically treated patients with PPF, the authors evaluated the functional outcome through the functional independence measure questionnaire and the modified Merle d'Aubigne and Postel score in addition to reporting on LLD and gait pattern [15]. We found that the presence of associated injuries and increased length of hospital stay negatively affected the functional outcomes. Although the fracture pattern did not significantly affect the functional outcomes, the least scores were obtained in patients with vertical shear fractures.

Regarding radiographic outcomes, although the vertical displacement did not show a statistically significant change post-operatively, the reduction quality in all patients was graded as excellent or good according to Matta and Tornetta’s grading system. Furthermore, the improvement in the symphyseal diastasis was not significant post-operatively; this was explained because some patients had a lateral compression injury that led to symphysis pubis overlapping; hence, the gap increased after reduction. We obtained a mean residual pelvic asymmetry of 0.83 ± 0.7 cm; however, it did not affect the patients’ functional outcomes, which was consistent with the results of the study by Oransky et al. [30], who reported various degrees of pelvic asymmetry in three (37.5%) of the surgically treated patients from the total of eight patients. They considered the asymmetry significant in two of the three patients. However, the authors reported that this was without clinical consequences. Conversely, some authors [1, 29, 30, 36–38] considered a residual asymmetry of the hemipelvis as > 1 cm a potential functional disability, with possible consequences of low back pain and limb shortening, sacroiliac pain, and nonstructural scoliosis. However, we did not encounter an adverse effect of residual asymmetry in our patients, which could be attributed to the relatively short follow-up and the ability of the paediatric patient to compensate with some degrees of deformity.

We reported complications in 21 (46.7%) patients, and none of them occurred intra-operatively. Approximately half were a pin tract infection after the external fixator application, which was conservatively treated and completely resolved following implant removal. In contrast, Zhu et al. reported a pin tract infection occurring in 7.5% of their series [11]. Furthermore, six patients had a residual vertical migration resulting in LLD, which was < 1 cm and was tolerated by the patients. In the study by Salášek et al. [8], the incidence of overall complications was 7.3%, which commonly included pelvic asymmetry, neurological deficits, nonunion, and ectopic calcification; they reported that the incidence of the complications was significantly higher with types B and C (*p* = 0.0015) and surgical management (*p* < 0.0001). In the systematic review by Sridharan et al. [9], the most commonly occurring early complications were infection (5%), followed by hardware-related complications necessitating removal (2.9%). In comparison, the most commonly occurring late complications were pelvic ring asymmetry (9.2%), followed by limping gait (6%), and LLD (5%).

Another controversial point related to PPF is the need for implant removal owing to the fear of growth arrest and premature permanent SIJ closure. In the current study, all applied external fixators were removed, and further hardware removal was performed in four patients (prominent metals in two and ISS in the other two, of whom one was infected). We avoided hardware removal unless needed or upon request from the patients’ parents. Galos and Doering reported that they routinely removed the external fixators by eight weeks and the ISSs by six months [14]; Oransky et al. [30] also recommended removing all implants between three and four months post-operatively to avoid growth arrest. Fahmy and Abdelmoneim reported that they scheduled plate and screw removal in their patients during the latest follow-up visit; however, they did not report hardware removal data indications or obstacles encountered [15]. In contrast, Kruppa et al. [7] selectively removed hardware as per patients’ request; they removed the hardware from only 3 of 16 patients (ISS, 3; symphyseal plate, 1) following fracture healing. Guimaraes et al. [39], who treated 13 patients with cannulated ISS to stabilize sacroiliac dislocation, reported that they did not remove any implants following fracture healing.

Our study has some limitations. First is the relatively small sample size; however, the number of patients with surgically treated PPF in the current study is considered comparable with or even more than most of the previous reports, which ranged from one to 41 patients with surgically treated PPF [1, 7, 30, 35–39]. This also has affected the robustness of evaluating the effect of various factors on the outcomes after dividing patients into groups based on their age and length of hospital stay, which needs further evaluation. Second is the relatively short follow-up period considering that more time is needed to determine the long-term complications with further growth. Third, we found difficulties in comparing the functional outcomes with previous reports due to different measuring tools; however, we hope that the modification we introduced to the Majeed functional scoring system will be widely adopted for evaluating patients with PPF. Fourth is the possible errors in radiographic measurements, considering that they were performed by one of the authors; hence, it lacks inter-and intra-observer reliability testing, and patients’ positioning could affect the measurements. However, the methods we used for radiographic assessment were used in some previous studies [7, 30, 39]. Fifth, fallacies in assessing the symphyseal diastasis are probably due to deficient standard measurements as the width of the symphysis changes significantly with age. Lastly, we did not profoundly investigate various factors affecting the outcomes due to the relatively small number of included patients as well as we did not compare the results with another cohort of patients treated nonsurgically.

## Conclusions

We achieved acceptable short-term functional and radiological outcomes in the current series after surgically treating a group of paediatric patients with PPF; however, the effect on the long-term outcomes still to be determined. The proper approach and fracture fixation tool should be tailored according to, patient characteristics, the fracture classification, and the presence of associated injuries.

## Supplementary Information

Below is the link to the electronic supplementary material.

Supplementary file1: Our trauma unit fractured pelvis and acetabulum registry form sheet. (PDF 571 KB)

## Data Availability

All the data related to the study are mentioned within the manuscript; however, the raw data are available with the corresponding author and will be provided upon a written request.
