# Supplementary material for: Surgical management of paediatric pelvic fractures: a prospective case series and early experience from a level one Egyptian trauma centre
Source: Int Orthop. 2022 Jul 23;46(10):2315–28. doi: 10.1007/s00264-022-05509-8 (PMC9492609; doi:10.1007/s00264-022-05509-8)
Supplement: Supplementary file 1 — Supplementary file1: Our trauma unit fractured pelvis and acetabulum registry form sheet. (PDF 571 KB) [file 264_2022_5509_MOESM1_ESM.pdf]

Assiut University Hospital

Fracture Pelvis Registry Form

Personal data

Name: **age:** **sex: 1(M) 2(F)**

Address:

Occupation:

Marital status:

Telephone number:

Date of trauma: Date of admission:

Date of discharge: Hospital no:

Comorbidity: Diabetes Hypertension Cardiac Renal Hepatic

Mechanism of Trauma:

☐ MCA ☐ Train Accident ☐ Motorcycle Accident ☐ FFH ☐ FOG

☐ Sharp object ☐- Others ☐- Heavy object

Fracture Pelvic Ring

- **Affected side :** ☐- **RT** ☐- **LT** ☐- **Both**

Classification:- ☐ **Closed** ☐- **Open:** ☐ **I** ☐ **II** ☐ **III**

*** According to Mechanism of Injury (Young & Burgess):**

1- AP Compression :

☐ **I**
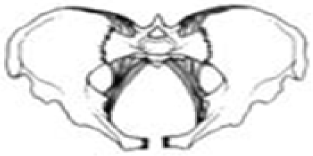

☐ **II**
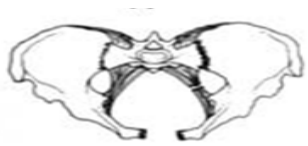

☐ **III**
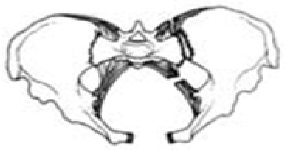

Sp diastasis < 2.5 cm **Sp diastasis > 2.5 cm** **complete disruption**

2-Lateral Compression:

☐ **I**
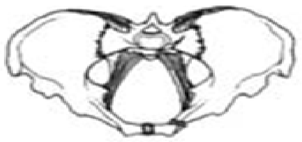

II ☐
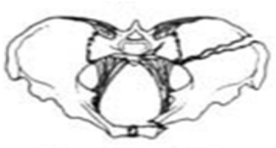

☐ **III**
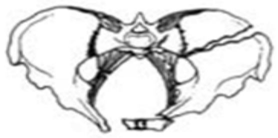

Ipsilateral Sacral Compression **Ipsilateral crescent f** **I /II+ Contralateral open book**

3-Vertical Shear:

☐ **I**
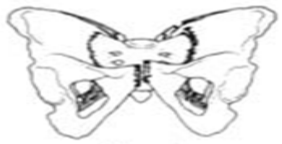

☐ **II**
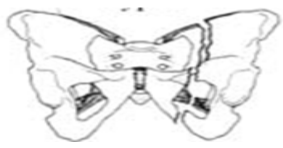

☐ **III**
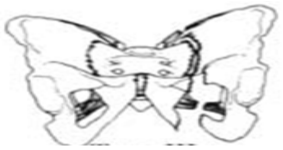

4-Combined Mechanism:

☐ **Anterolateral Force**

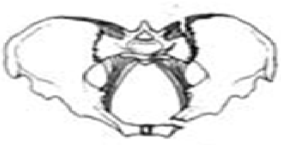

☐ **Antero vertical Force**

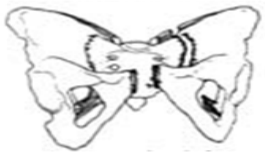

<u>* According to pelvic Stability (Tile's):</u>

A: - Stable:

☐ **A 1** — **Avulsion innominate bone**

☐ **A 2** — **non/ minimal displaced**

☐ **A 3** — **sacral/coccyx transverse**

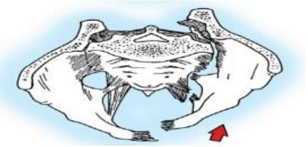

B - Partially stable (rotationally unstable, vertically stable):

☐ **B1** — **Open book**

☐ **B 2** — **Lateral compression**

☐ **B 3** — **bilateral injury**

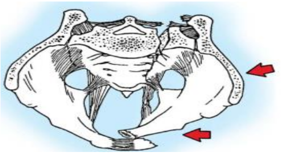

C:-unstable (Both rotationally & vertically):

☐ **C 1** — **Unilateral**

☐ **C 2** — **Bilateral (B& c)**

☐ **C 3** — **Bilateral c**

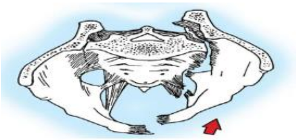

B – Fracture Acetabulum

<u>Type of Trauma:</u> ☐ **Direct** ☐ **Indirect**

<u>Affected Side:</u> ☐ **RT** ☐ **LT** ☐ **Both**

<u>Classification</u> ☐ **Closed** **Open:** - ☐ **I** ☐ **II** ☐ **III**

<u>Judet – Letournel classification:</u>

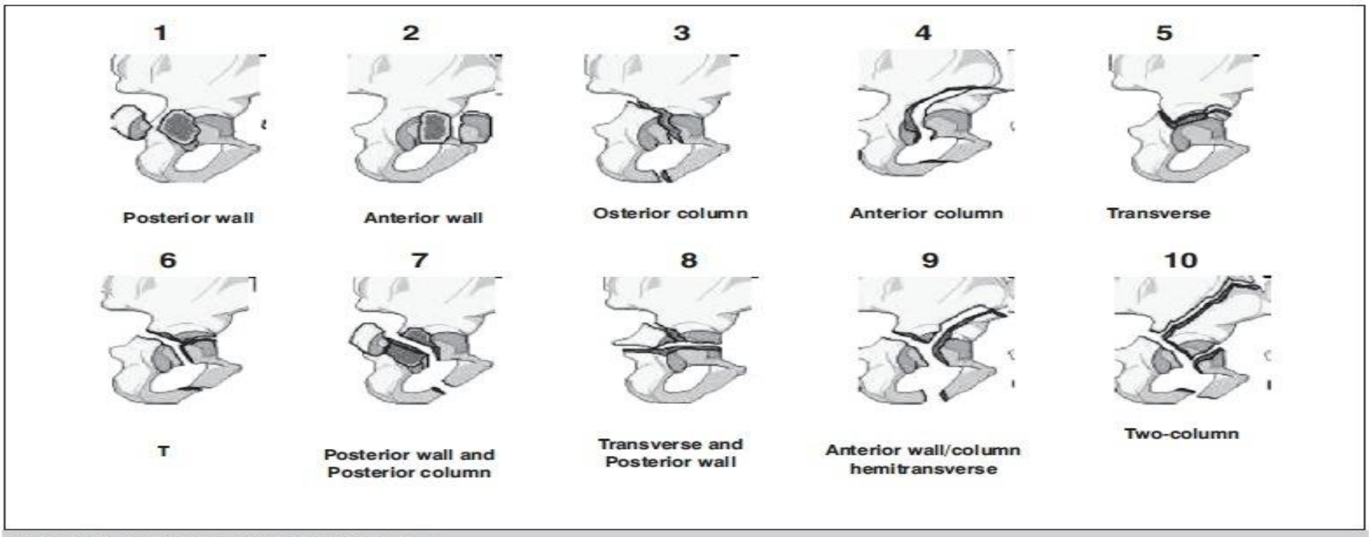

Figure 1. *Classification of Judet and Letournel.*

☐ **Posterior wall** ☐ **Anterior wall** ☐ **posterior column** ☐ **anterior column**

☐ **Transverse** ☐ **T** ☐ **posterior wall and posterior column**

☐ **Transverse and posterior wall** ☐ **anterior wall/column hemi transvers** ☐ **Two-column**

AO classification

A - Only one column involved

☐ **A1** 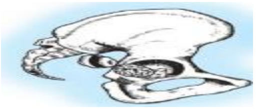 ☐ **A2** 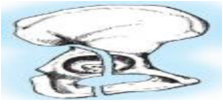 ☐ **A3** 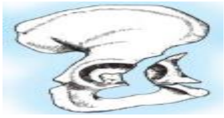

B-Transverse Fracture, portion of roof remains attached to ilium :

☐ **B1** 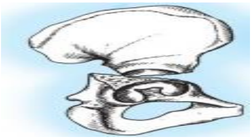 ☐ **B2** 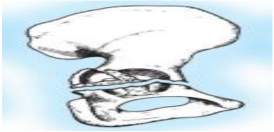 ☐ **B3** 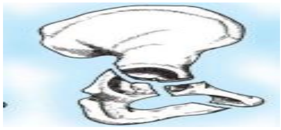

C: Both columns involved

☐ **C1** 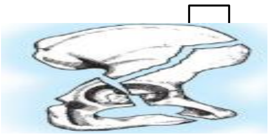 ☐ **C2** 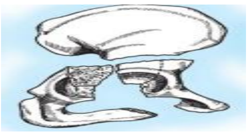 ☐ **C3** 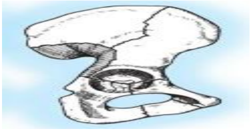

C-Sacral classification

☐ Unilateral ☐ Bilateral

Dennis classification

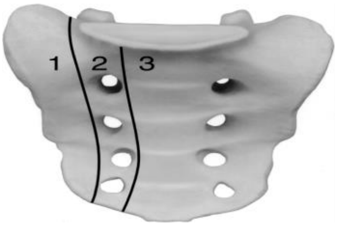

☐ I ☐ II ☐ III

Transverse sacral fracture(type III but the fr line tansverse the 3 zones)

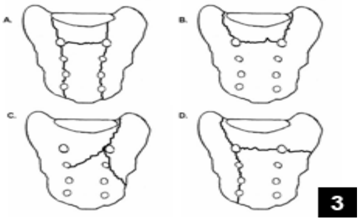

☐ H ☐ U ☐ lambda ☐ Y

Roy-Camille/Strange-Vognsen and Lebech Classification of Transverse Sacral Fractures

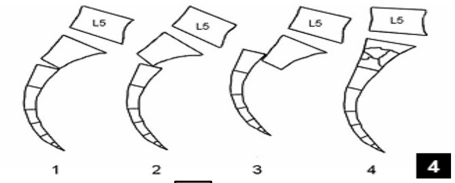

☐ I ☐ II ☐ III ☐ IV

CT evaluation

Intra-articular loose fragments **Marginal impacted** **Fracture Commination**

Rotation of fracture fragments **Femoral head lesions** **Joint Congruence**

SIJ and the posterior pelvic ring

Treatment

☐ **Non operative:**

☐ **Operative:** ☐ **External Fixation** ☐ **Iliosacral screw** ☐ **iliac plate** ☐ **iliac lag screw** ☐ **lag screw**

☐ **Symphysis plate** ☐ **sacral tension band plate** ☐ **Anterior brime plate** ☐ **LC2 screw**

☐ **One posterior acetabular plate** ☐ **Anterior infrapectinal plate** ☐ **supraacetabular screw**

☐ **Quadrilateral buttress plate** ☐ **Two posterior acetabular plate**

☐ **Anterior column lag screw:** **1-Antegrade** **2-Retrograde**

☐ **Posterior column lag screw:** **1-Antegrade** **2-Retrograde**

Operative Approaches:

☐ **Posterior (Kocher – Langenbach)** ☐ **pfannenstiel incision** ☐ **Ilioinguinal**

☐ **Modified stoppa** ☐ **Pararectus** ☐ **Minipararectus** ☐ **surgical dislocation**

☐ **Iliofemoral (smith Peterson)** ☐ **Extended Iliofemoral**

Associated Orthopedic Injuries:-

1-Upper limb: a- clavicle b-scapula c- humers d-radius e- ulna

f- Carpal bone g- metacarpal h-phalanges

2- Lower limb: a-trochanteric femur b- neck femur c-head femur e- Shaft femur f-supracondylar femur

G-Tipial plateau h- shaft tibia j- ankle k - Metatarsal

3- Spine: a-cervical vertebra b-dorsal vertebra c- lumber vertrbra

Treatment: non-operative operative

Associated Injuries:-

1. Neurological:

☐ **Lumbosacral plexus** ☐ **Sacral Roots** ☐ **Sciatic N palsy**

2-Genitourinary:

☐ **Urethra** ☐ **Bladder: a- Extra peritoneal b- Intraperitoneal**

☐ **Scrotum** ☐ **vagina**

***Treatment: 1-Non operative 2-operative**

3. Gastrointestinal:

☐ **Rectum** ☐ **perianal tear** ☐ **Intestinal** ☐ **Liver** ☐ **Spleen** ☐ **IPH**

***Treatment: 1-Non operative 2-operative**

4. Head injuries:

GCS: Severe 3-8 Moderate 9-12 Minor 13-15

a- Fracture skull b-Brain contusion c- brain edema d- subarachnoid hemorrhage

e-intracranial hemorrhage f- Extradural hemorrhage j-cerebral hemorrhage

Treatment: 1-Non operative 2-operative

5. Chest injuries:

A -fracture ribs b-heamothorax c-pneumothorax E -lung contusion f- flail chest

Treatment: 1-Non operative 2-operative

Injury severity score (ISS)

Iss: <20 **25-40** >40

Pre-operative medication

1-anticoagulant drug **: LMWH Aspirin warfarin (marevan)**

2-corticosteroid

Intraoperative data

Date of operation:

Position: a-supine b- prone c-lateral

Anesthesia: a-spinal b-epidural c-general

Time: Blood loss: blood transfusion:

Soft tissue injury: **Tscherne Classification of Closed Fractures**

Grade 0 **I** **II** **III**

Suction drain: a-yes b-No

Implant used: a- narrow plate b-reconstruction 4.5 c- Reconstruction 3.5 d-small plate

f- Lag screw g-other h-locked plate

Difficulties at the operation:

Complication: sciatic nerve palsy vascular injury Infection

Other operations:

Image shots:

Surgeon: Assistant surgeon:

Antibiotic type: Antibiotic dose:

Post-operative assessment

Post-operative hospital stay:

DVT Infection Bleeding

A- Radiological

1- Matta and tornetta grade pelvis:

Excellent: <4mm

Good: 4–10mm

Fair: 10–20mm

Poor: >20mm.

2- Matta of acetabulum:

1- Anatomical 0-1mm displacement

2- Imperfect 2-3mm

3- Poor >3mm

B-Neurological Ex.

Follow up

Radiological:

1- Union non- Union mal –Union

3- Excellent grade: normal appearing hip joint

Good: minimal sclerosis and joint narrowing

Fair: moderate sclerosis and joint narrowing (\50 %)

Poor: greater changes. At the final follow-up,

Clinical:

1-harris hip score:

Excellent result: 90-100 points
Good result: 80-89 points
Average earnings: 70-79 points
Bad result : <70 points

2-mageed score:

working before injury	Non-working before injury
Excellent: ›85	Excellent : ›70
Good: 70-84	Good: 55-69
fair: 55-69	fair: 45-54
poor: <55	poor: <45
